# Cost-effectiveness of microendoscopic discectomy versus conventional open discectomy in the treatment of lumbar disc herniation: a prospective randomised controlled trial [ISRCTN51857546]

**DOI:** 10.1186/1471-2474-7-42

**Published:** 2006-05-13

**Authors:** Mark P Arts, Wilco C Peul, Ronald Brand, Bart W Koes, Ralph TWM Thomeer

**Affiliations:** 1 Department of Neurosurgery, Leiden University Medical Center, Leiden, The Netherlands; 2 Department of Neurosurgery, Medical Center Haaglanden, The Hague, The Netherlands; 3 Department of Medical Statistics, Leiden University Medical Center, Leiden, The Netherlands; 4 Department of General Practice, Erasmus Medical Center, Rotterdam, The Netherlands

## Abstract

**Background:**

Open discectomy is the standard surgical procedure in the treatment of patients with long-lasting sciatica caused by lumbar disc herniation. Minimally invasive approaches such as microendoscopic discectomy have gained attention in recent years. Reduced tissue trauma allows early ambulation, short hospital stay and quick resumption of daily activities. A comparative cost-effectiveness study has not been performed yet. We present the design of a randomised controlled trial on cost-effectiveness of microendoscopic discectomy versus conventional open discectomy in patients with lumbar disc herniation.

**Methods/Design:**

Patients (age 18–70 years) presenting with sciatica due to lumbar disc herniation lasting more than 6–8 weeks are included. Patients with disc herniation larger than 1/3 of the spinal canal diameter, or disc herniation less than 1/3 of the spinal canal diameter with concomitant lateral recess stenosis or sequestration, are eliglible for participation. Randomisation into microendoscopic discectomy or conventional unilateral transflaval discectomy will take place in the operating room after induction of anesthesia. The length of skin incision is equal in both groups. The primary outcome measure is the functional assessment of the patient, measured by the Roland Disability Questionnaire for Sciatica, at 8 weeks and 1 year after surgery. We will also evaluate several other outcome parameters, including perceived recovery, leg and back pain, incidence of re-operations, complications, serum creatine kinase, quality of life, medical consumption, absenteeism and costs. The study is a randomised prospective multi-institutional trial, in which two surgical techniques are compared in a parallel group design. Patients and research nurses are kept blinded of the allocated treatment during the follow-up period of 2 years.

**Discussion:**

Currently, open discectomy is the golden standard in the surgical treatment of lumbar disc herniation. Whether microendoscopic discectomy is more cost-effective than unilateral transflaval discectomy has to be determined by this trial.

## Background

Sciatica due to lumbar disc herniation refractory to conservative treatment is effectively treated by surgery. The primary goal of surgery is retrieval of herniated disc fragments and decompression of the nerve root. Since the first report of lumbar disc surgery in 1934 by Mixter and Barr [1], who performed a laminectomy with transdural disc removal, various less invasive techniques have been developped. With the introduction of the microscope, Yasargil and Caspar refined the original laminectomy into the open microdiscectomy [2,3]. This technique has become the most common procedure worldwide. In 1997 Foley and Smith introduced the transmuscular approach of microendoscopic discectomy (MED) with advanced optics and instruments applicated in laparoscopic surgery [4]. Later, the original endoscopic procedure was modified with the operative microscope which has led to the development of the Microscopic Endoscopic Tubular Retractor System (METRX). This technique is subject of our protocol.

The concept of minimally invasive spine surgery is less tissue damage, while achieving good clinical outcome comparable with conventional open surgery. Patients are expected to have less back pain, shorter hospitalisation and quicker resumption of daily activities. Moreover, the cost-effectiveness is expected to be superior.

The Cochrane review of lumbar disc surgery has shown considerable evidence on the effectiveness of discectomy in patients in whom conservative management has failed [5]. Three studies compared microdiscectomy versus standard open discectomy [6-8]. Use of the microscope lengthened the surgical procedure but there was no significant difference in clinical outcome. The expected earlier return to work was not realised. This could be explaned by the rather small difference in invasiveness between microdiscectomy and the frequently performed unilateral transflaval approach by using loupe magnification. Nowadays, these two procedures are lumped together. A randomised controlled trial (RCT) between microdiscectomy with microscope versus microdiscectomy with loupe magnification has not been performed.

The Cochrane review did not include trials concerning MED. However, MED has proven it's safety and efficiency in multiple studies [4,9-15]. Three of these studies have compared MED with conventional open discectomy [10,12,13], 1 was randomised [12]. There is a trend towards faster recovery and less tissue damage in MED, although due to limited number of patients and methodological flaws, no firm evidence based conclusions can yet be drawn.

The rational of MED is a muscle-splitting approach by using sequential dilators and insertion of a tubular retractor, instead of subperiostal muscle dissection in the conventional open procedure. Iatrogenic devascularisation and denervation of paraspinal muscles is regarded to be one of the causes of poor clinical outcome and failed-back-surgery syndrome [16]. Therefore, the muscle-splitting technique of MED is expected to result in less tissue damage since subperiostal muscle dissection is prevented. However, 1 study of 40 patients failed to show a significant difference in post-operative contrast enhanced magnetic resonance imaging (MRI) with respect to paraspinal muscle damage between MED and conventional open discectomy [11].

Another parameter to analyse post-surgery tissue damage is serum creatine kinase (CK) as indicator of muscle injury. CK increases after spinal surgery and reaches a maximal value 1 day after surgery [17]. Nakagawa et al. have compared CK values on day 1 after MED and conventional open discectomy. They showed a significant difference in postoperative CK in favor of the MED [10]. Whether this is related to clinical outcome is not known.

Minimally invasive spine surgery has limitations as well. One of the disadvantage of MED is potential nerve root injury because of limited exposure. On the contrary, it is shown that MED causes less intraoperative nerve root irritation compared to conventional surgery [12].

Another issue related to limited exposure is recurrent disc herniation. The recurrence rate after MED is not known but it is expected to be higher than after conventional open discectomy since less disc material is retrieved. However, a recent randomised trial has shown superiority of sequesterectomy only, compared to conventional discectomy with regard to recurrence rate [18]. Therefore, the relationship between the extent of disc retrieval and recurrence rate is debated. In our study we will weight the total amount of disc retrieval in both surgical techniques and correlate this with disc recurrency.

Like other new minimally invasive techniques, MED has a learning curve which is related to surgery time, complications, conversion to the open procedure, and recurrent disc herniation. It is demonstrated that a surgeon should perform at least 30 procedures in order to be skilled and know the pitfalls [14]. Skill acquisition of MED is necessary before clinical assessment of MED versus conventional open discectomy can be started. Therefore, in our trial we have selected surgeons with large experience in MED.

Presently, the golden standard in surgical treatment of lumbar disc herniation is the unilateral transflaval discectomy to which all other techniques should be compared. The purpose of our study is to assess whether MED is more (cost)-effective than conventional open discectomy. The cost-effectiveness results will be a trade-off between a quicker relief in leg pain in the MED group versus the advantage of lower costs in the conventional group. Moreover, we will identify possible subgroups of patients who will substantially benefit from one of the allocated surgical treatments. Since MED is hypothesised to have a particularly favorable short term effect, we will set 8 weeks and 1 year as primary measure points.

## Methods/Design

We designed an observer and patient blinded randomised cost-effectiveness trial in the treatment of lumbar disc herniation in which two surgical techniques are compared in a parallel group design. The primary outcome measure is the Roland Disability Questionnaire for Sciatica. The follow-up period will last 2 years. In order to collect enough patients, a multi-center design is necessary. The study protocol was approved in all participating hospitals.

Our primary question is whether MED is more (cost)-effective than open discectomy. The analysis will be focused on the proportion of patients who are recovered to a normal functional situation at the short and long term. Moreover, we want to identify certain subgroups who may benefit more of one of the allocated treatments.

### Patients

**Figure 1:**
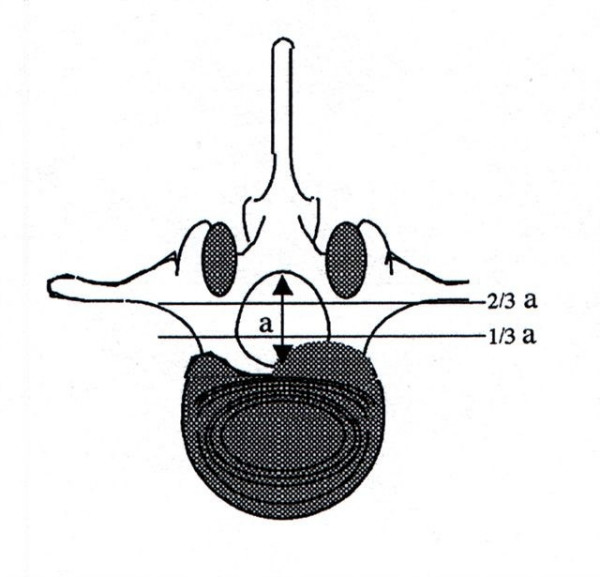
The size of the herniated disc in relation to the spinal canal diameter is measured at disc level.

All patients between 18 and 70 years with sciatica lasting more than 6–8 weeks are eligible for this study. Imaging studies (MRI) must confirm a herniated disc larger than 1/3 of the spinal canal diameter (figure 1), or a smaller herniated disc with concomitant lateral recess stenosis or sequestration. Additional inclusion and exclusion criteria are listed in table 1. Patients with small herniated discs less than 1/3 of the spinal canal diameter are eligible for our parallel study of percutaneous laser discus decompression (PLDD) which compares PLDD with conventional discectomy. This trial will be discussed seperately.

Patients are referred by a neurologist with MRI and conventional imaging of the lumbar spine. During the first visit to the neurosurgical outpatient clinic, the patient's history and a standard neurological examination will be documented. Conform our selection criteria, the neurosurgeon decides whether a patient is eligible for the MED trial. The study will be explained to the patient and, in case of a positive reaction, an appointment is made with one of the research nurses. Because the patient needs some time to consider participation, the first visit to the research nurse is planned after at least 2 days. After informed consent, the questionnaires, outcome measures and baseline variables are recorded.

### Randomisation procedure

Patients will be randomly allocated to either MED or conventional discectomy. Randomisation will take place on the operating room within 4 weeks after the first visit to the research nurse. A randomisation list is prepared for every participating hospital-nurse combination. Variable-sized blocks of random numbers are formed to ensure equal distribution of the randomisation treatments over hospitals and research nurses. The data manager at the department of Biostatistics, who is not involved in the selection and allocation of patients, will prepare coded, sealed envelopes containing the treatment allocation. In the operating room, after induction of anesthesia, the surgeon will open the envelope and the allocated treatment is performed. Patients and research nurses are kept blinded for the allocated treatment during the follow-up period of 2 years.

### Intervention

Patients will be randomised into conventional unilateral transflaval discectomy (A) or MED (B). Depending on the surgeon and patient's preference, surgery will be performed under general or spinal anesthesia. The patients will be positioned prone and the affected disc level is verified with fluoroscopy. The participating surgeons have large experience in both techniques. A standardized Case Record Form (CRF) will register the surgeon's findings and this CRF, together with the randomisation envelope, will be returned to the data center in a sealed envelope.

### (A) Unilateral transflaval discectomy

A small midline incision (2–3 cm) will be made and the paravertebral muscles will be discected unilaterally. Laminotomy will be performed when necessary. In order to decompress the nerve root, the herniated disc will be removed as much as possible through a unilateral transflaval approach. All retrieved disc material will be collected and weighted afterwards. The wound will be closed in layers with a suction drain when necessary. Patients will be operated with loupe magnification or microscope depending on the surgeon's preference.

### (B) Microendoscopic discectomy

The same size midline incision will be made, the skin will be retracted laterally and the guidewire and sequential dilators will be placed paraspinal under lateral fluoroscopy control. The tubular retractor will be connected to a flexible arm and fixed. Under microscopic view a unilateral flavectomy and discectomy will be peformed. All removed disc material will be collected. The wound will be closed in layers with a suction drain when necessary. Whenever MED will be converted to an open discectomy, the patient will maintain in group B conform the 'intent to treat'principle.

Surgery will take place within 4 weeks after inclusion on the outpatient neurosurgical clinic. Hospital admission will be 2–7 days (including the day of admission), depending on the usual care of the participating hospital. During the immediate post-operative period the patients will be mobilised as soon as possible with the help of a physiotherapist. Attempts will be made to discharge the patient as soon as possible in both study groups. The physician who will discharge the patient is not aware of the performed treatment. Patients and their guided physiotherapists are stimulated to resume homeactivities and work as soon as possible.

### Baseline data

The baseline questionnaire assesses demographics, hobbies, sports, work status, smoking status, back pain history, family history of sciatica, co-morbidity, weight and length. The patient's satisfaction at work will be registered. The treatment preference of both patient and surgeon will be assessed on a 5-point scale ranging from "strong preference for conventional open discectomy" to "strong preference for MED". Moreover, the expected recovery 8 weeks after surgery of both patient and surgeon will be registered.

### Outcome assessment

We will use the below described validated outcome parameters which will be assessed by means of questionnaires. Follow-up examinations by the research nurse will take place 4, 8, 26 and 52 weeks after randomisation. Patients will be neurologically examined and questionnaires will be filled in (table 2). In between at 1, 2, 6, 12, 38, 78 and after 104 weeks the main questionnaire (primary outcome measures) will be filled in at home and send to the data center. The outpatient control by the neurosurgeon will be at 8 weeks and more if necessary.

We hypothesise a difference in (cost)-effectiveness between MED and conventional discectomy in favor of the MED on the short term, and comparable (cost)-effectiveness after 6–12 months. Therefore the main questionnaire will be answered at 8 weeks and 1 year after surgery.

#### Primary outcome measure

Roland Disability Questionnaire for Sciatica (RDQ). This illness-specific 23 item functional assessment questionnaire is frequently used for low back pain and sciatica [19-22]. Scores range from 0 to 23, reflecting a simple unweighted sum of items endorsed by the respondent. Patients with high scores at baseline have a severe disabling sciatica. To define recovery, a difference of at least 11 points from baseline has to be seen. This self report measure of physical disability due to back and leg pain has established validity, reliability, and responsiveness to change [23].

#### Secondary outcome measures

##### 1) Perceived recovery

This is a 7-point Likert scale measuring the perceived recovery, varying from 'complete recovery' to 'worse than ever'. This outcome scale has been used in previous studies and is regarded valid and responsive to change [24]. Next to this global selfassessment, a job and hobby specific Likert will be scored. During the intake of the study the patient will be asked to rank their 5 most important functional disabilities in daily life, which they can use in their own evaluation overall and in separate items.

##### 2) Visual Anolog Scale (VAS) of leg pain

This parameter will measure the experienced pain intensity in the leg during the week before visiting the research nurse. Pain will be assessed on a horizontal 100 mm scale varying from 0 mm, 'no pain', to 100 mm, 'the worst pain imaginable'. Patients do not see the results of earlier assessments and will score the pain experienced at the visit. Reliability, validity and responsiveness of VAS have been shown [25].

##### 3) VAS of back pain and combined leg and back pain

Since many patients with sciatica have back pain as well, we will also measure the intensity of solitary back pain and the combination of leg and back pain. There may be a difference in back pain between the muscle-splitting microendoscopic technique and the conventional muscle-discecting technique.

##### 4) McGill pain questionnaire

Several qualities of perceived pain will be measured using the Dutch version of the McGill Questionnaire [26].

##### 5) Short-form 36 (SF 36)

This is a generic health status questionnaire which can easily be filled out at home. The questionnaire consists of 36 items on physical and social status of the patient subdivided in 8 domains; 1) physical functioning, 2) physical restrictions, 3) emotional restrictions, 4) social functioning, 5) somatic pain, 6) general mental health, 7) vitality and 8) general health perception. The questions are scored on a scale of 0 (worst health) to 100 (ideal health). This questionnaire has been used frequently and is validated in surgical studies on low back pathology [27-29].

##### 6) Sciatica Frequency and Bothersome Index (SFBI)

This is a scale ranging from 0 to 6, which can assess the frequency (0 = never to 6 = always) and bothersome (0 = not at all to 6 = extreme bothersome) of back and leg pain. The sum of the results of four symptom questions yields both indexes, ranging from 0 to 24. The four questions are: leg pain, numbness and/or tingling in leg, weakness in leg or foot, and pain in back or leg while sitting [19,30].

##### 7) Prolo scale

This scale measures the evaluation of the surgeon and research nurse of the patient's functional and economic status. It has been used in outcome studies of lumbar spinal operations [31].

#### Other outcome measures

##### 1) Costs

The direct medical costs of hospital admission and surgery will be based on an integral cost-analysis in three regional participating hospitals. From this institutional analysis, the constant costs per admission and the variabel costs per admission day will be estimated. Knowing the duration of hospitalisation, the individual costs of all operated patients can be estimated. Other medical costs (including physiotherapy, visits to general practioners and medical specialists, nursing care and medication) will be registered in a diary. In the diary the patient will also register non-medical costs (including time costs, travel expenses and domestic help). The research nurse will go through the diary with the patient on every follow-up moment throughout the study period of 2 years. To estimate the indirect costs, like productivity costs, patients will register absenteeism in the diary and the research nurse will register the patient's work situation, work efficiency and gross income on follow-up moments. Absenteeism will be valued to the friction-cost method.

##### 2) Incidence of re-operations

In general, re-operation is considered as bad outcome and therefore used as an outcome measure. The incidence of re-operation in both groups will be measured.

##### 3) Complications

A systematic assessment of complications (including wound infection, deep venous thrombosis, urine tract infection, hematoma, and progressive neurological deficit) will be carried out by the surgeon and research nurse. Moreover, surgeons will be asked for perioperative complications like cerebrospinal fluid leakage, nerve root damage, and exploration on the wrong disc level.

##### 4) Serum creatine kinase (CK)

Creatine kinase is a known marker of muscle damage. The post-operative serum level of CK has a maximal value on day 1 after spinal surgery [17]. Therefore, we will determine the serum CK before surgery and 1 day after surgery in both groups to assess a possible correlation between CK and extent of surgery, i.e. transmuscular versus muscle discection. Whether CK is correlated with back pain and recovery will also be determined.

### Sample size

The sample size is calculated on the basis of the Roland Disability Questionnaire for Sciatica. A difference in effect between MED and open discectomy is assumed whenever MED will obtain at least 20% difference in Roland score at 8 weeks after randomisation. Conventional open discectomy is assumed to have at least 60% good result 8 weeks after surgery. This means that MED is proven to be more effective than conventional surgery whenever 80% or more of the patients have a good outcome 8 weeks after surgery. The numbers used for this sample size are drawn from the Maine Lumbar Spine Study 1 year and 5 year results and were discussed in detail recently [19,21].

This trial attempts to enroll 300 patients with lumbar disc herniations (150 patients in both treatment groups, including 8% loss to follow-up) which is regarded sufficient to detect a difference of 20% (α = 0,05 and a power of 90%) in the primary outcome.

### Statistical analysis

Baseline comparability will be analysed by descriptive statistics to determine whether randomisation was successful. Whenever necessarry, adjustments for baseline variables will be performed in the analysis. Differences in outcome measures between both groups, together with 95% confidence intervals, will be calculated. Besides a difference in recovery between the two groups, analysis of a difference in time to recovery will be carried out as well. All data are analysed according the "intent-to-treat principle". In addition, an explorative subgroup analysis is conducted to investigate whether the treatment effect varies over specific subgroups of patients (table 3). Data will be stored via the internet-based secure data management system "ProMISe" of the department of Medical Statistics and Bioinformatics. The analyses will be carried out using appropriate statistical software (e.g. SPSS).

## Discussion

In this article we introduce a design of a RCT on the (cost)-effectiveness of MED versus conventional unilateral transflaval surgery in the treatment of lumbar disc herniation. This is the first randomised prospective trial comparing these two surgical techniques. Moreover, the study is unique in the way that patient and research nurse are blinded for the allocated treatment. The objective of this trial is to determine whether the minimally invasive surgical technique of MED is more (cost)-effective than conventional unilateral transflaval surgery. The inclusion period will run until the end of 2006 and follow-up measurements will be completed in the end of 2008.

## Abbreviations

MED: Micro Endoscopic Discectomy

RCT: Randomised Controlled Trial

VAS: Visual Analogue Scale

CRF: Case Record Form

RDQ: Roland Disability Questionnaire

CK: Creatine Kinase

## Competing interests

The author(s) declare that they have no competing interests.

## Authors' contributions

MA is the coordinator and principal investigator of the trial. WP designed the study protocol and is the supervisor of MA. RB has contributed to the case record forms and is responsible for the implementation of the trial data management using the ProMISe software. BK is the epidemiological supervisor. RT is the neurosurgical supervisor. All authors participated in the trial design and coordination. All authors read and approved the final manuscript.

## Pre-publication history

The pre-publication history for this paper can be accessed here:



## Figures and Tables

**Table 1:** Selection criteria for trial eligibility.

**Inclusion:**
• Age 18–70 years
• Persistent radiculair pain lasting more than 6–8 weeks
• Operation indication
• Disc herniation confirmed MRI
○ unilateral disc herniation larger than 1/3 of the spinal canal diameter or
○ unilateral disc herniation less than 1/3 of the spinal canal diameter with concomittant lateral recess stenosis or sequestration
• Informed consent
**Exclusion:**
• Previous lumbar surgery on the same disc level
• Cauda equina syndrome
• Spondylolytic or degenerative spondylolisthesis
• Central spinal canal stenosis
• Pregnancy
• Severe somatic or psychiatric illness
• Planned (e)migration to another country in the year after inclusion
• Inadequate knowledge of Dutch language

**Table 2:** Data collection and outcome measures.

Time in weeks	x	0	1	2	4	6	8	12	26	38	52	78	104
Treatment preference	v						v				v		v
Expected recovery at 8 weeks	v												
Likert	v	v	v	v	v	v	v	v	v	v	v	v	v
Neurological examination	v				v		v		v		v		
Prolo	v				v		v		v		v		
Severity of complaints (VAS)	v	v		v	v	v	v	v	v	v	v	v	v
McGill	v										v		v
Functional status (Roland)	v	v	v	v	v	v	v	v	v	v	v	v	v
Health status (SF 36)	v				v		v		v		v	v	v
EuroQol	v	v		v	v	v	v	v	v	v	v	v	v
SFBI	v				v		v		v		v		
Costs (diary)					v		v		v		v	v	v
Surgery		v											
CK		v											
Complications		v			v		v		v		v		

**Table 3:** Selected prognostic variables for subgroup analysis.

**Demographic variables:**
• Age < 40 years versus > 40 years
• Non-physical versus physical demanding jobs
**Anamnestic and neurological variables:**
• Influence of sitting on complaints versus no influenec
• Quetelet index < 25 versus > 25
• Straight leg raising < 30° without back pain versus with back pain
**Radiological variables:**
• MRI disc sequester versus contained disc herniation
• Median versus mediolateral and lateral disc herniation
• High versus low height of disc level
